# Case report: Endovascular treatment of acute limb ischemia in an adolescent with newly diagnosed lupus

**DOI:** 10.3389/fcvm.2023.1152929

**Published:** 2023-05-30

**Authors:** Lu-Hang Liu, Ming-Dar Lee, Ting-Huan Huang, Szu-Hung Chu, Mu-Yang Hsieh

**Affiliations:** ^1^ Division of Cardiology, Department of Pediatrics, Mackay Memorial Hospital, Hsin-Chu, Taiwan; ^2^ Division of Nephrology, Department of Pediatrics, Mackay Memorial Hospital, Hsin-Chu, Taiwan; ^3^ Division of Hematology, Department of Pediatrics, Mackay Memorial Hospital, Hsin-Chu, Taiwan; ^4^ Division of Immunology, Department of Pediatrics, Mackay Memorial Hospital, Hsin-Chu, Taiwan; ^5^ Cardiovascular Center, National Taiwan University Hospital Hsin-Chu Branch, Hsin-Chu, Taiwan; ^6^ Department of Biological Science and Technology, National Yang Ming Chiao Tung University, Hsin-Chu, Taiwan

**Keywords:** peripheral artery disease, acute limb ischemia, systemic lupus erythematosus, antiphospholipid syndrome, case report, adolecent

## Abstract

We report a 14-year-old adolescent who presented with acute limb ischemia caused by systemic lupus erythematosus-related antiphospholipid syndrome (APS). In the pediatric population, acute limb ischemia is rare. This case is unique in that after the initial medical treatment failed, interventional devices for acute stroke intervention were utilized to salvage the limb in our patient with a small tibial artery vessel to achieve procedural success. To provide limb salvage, operators may combine peripheral and neuro-intervention devices to maximize procedure success.

## Introduction

Acute limb ischemia is rare in adolescents. It can lead to lifelong disability and potential mortality when the underlying cause and anatomical obstruction are left unidentified, untreated, or under-treated (1). Here, we report a 14-year-old boy presented with acute limb ischemia of the left lower limb. The initial medical treatment failed to improve the distal limb perfusion. Then an invasive endovascular intervention utilizing the Penumbra aspiration system provided endovascular revascularization and successfully salvaged the left forefoot and toes. Systemic lupus erythematosus (SLE) with antiphospholipid syndrome (APS) was diagnosed by autoimmune markers in our case.

## Case presentation

**Figure 1:**
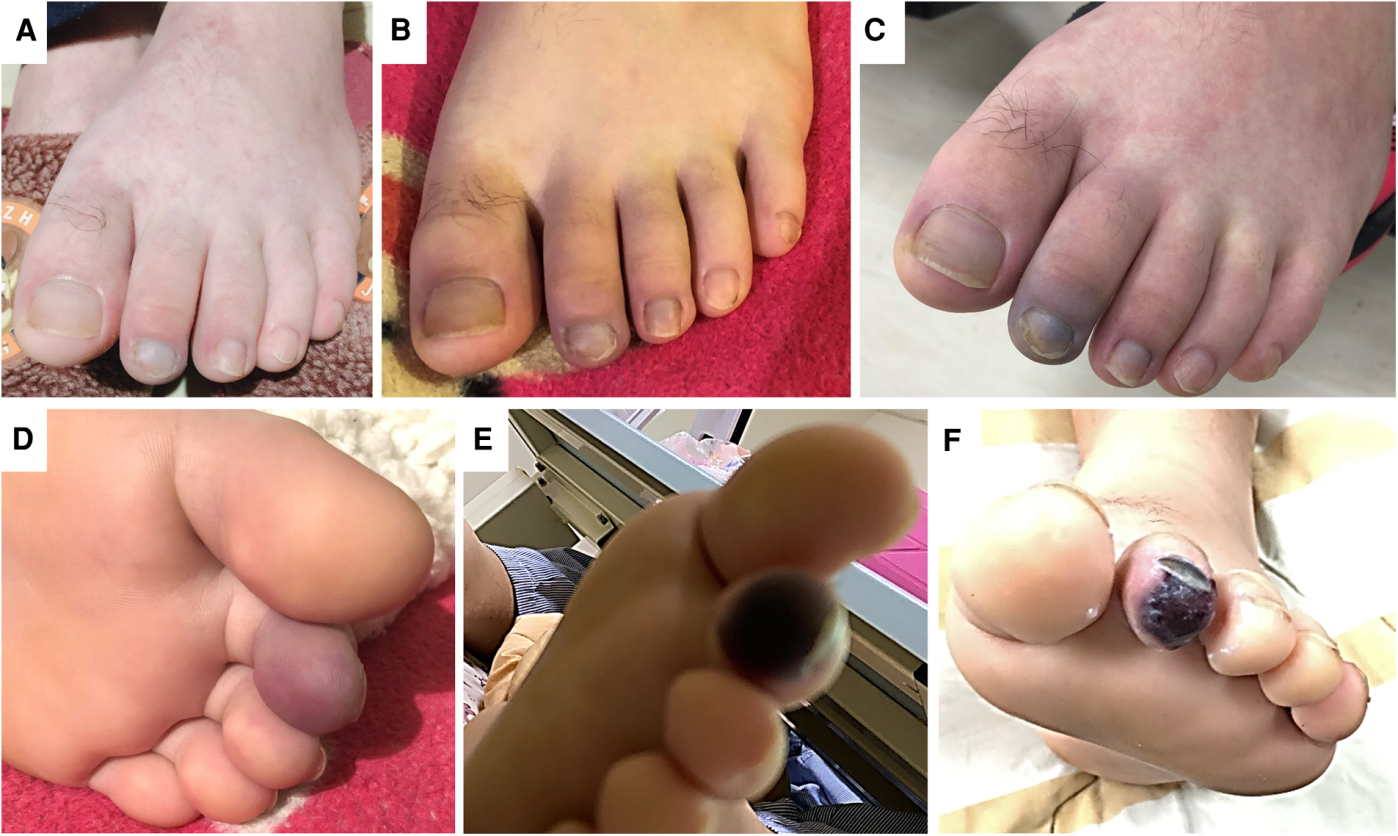
The physical findings of the left foot during the treatment course. (Panel **A**) Shows the photo on day 1. After commencing medical treatment, the pain persisted in the left toes and forefoot. (Panels **B**,**C**) Presents the skin color change on day 2 and 3. There was progressive cyanosis and impending gangrene change, as shown in (Panel **D,E**) by day 4. (Panel **F**) Shows the photo one week after the successful endovascular revascularization. The pain was relieved, and the gangrene was limited to the toe tip.

A 14-year-old boy presented with a 10-day history of left foot pain and swelling of his left anterior dorsal foot. His medical history included thrombocytopenia (platelet count 110 k/µl), found during a school survey one year before admission. There was no history of prior infection or recent vaccination, and the patient's presentation occurred months before the COVID-19 pandemic in our country. Besides the pain, his left second toe had black discoloration and localized tenderness. It was pale and cold on the left dorsal foot, with minimal pulsation over the dorsalis pedis artery (Figure 1). Laboratory results showed a C-reactive protein of 1.81 mg/dl and a D-dimer of 1,750 ng/ml. Vascular Duplex found loss of typical flow pattern of his left dorsalis pedis artery and anterior tibial artery. The flows in the ankle arteries were monophasic, and signals in the veins were still detectable. Moreover, he could still feel pain and move his ankle and toes freely. Acute limb ischemia in Rutherford stage I was diagnosed. Electrocardiography showed no atrial fibrillation, and the heart echo revealed no valvular vegetation or structural abnormalities. He denied photosensitivity, rash, or arthralgia. His mother has systemic lupus erythematosus diagnosed in young adulthood and received treatment accordingly. His autoimmune laboratory profile was abnormal, with a positive antinuclear antibody test, positive anti-double-stranded DNA test, positive lupus anticoagulant, abnormally low complement C3 and C4, and mild proteinuria. Therefore, the boy received a diagnosis of APS associated with SLE. He then received treatment with subcutaneous enoxaparin injection, prostaglandin E1 infusion, aspirin, prednisolone, and hydroxychloroquine. Due to the patient's relatively young age and the limited extent of cyanosis, invasive angiography was not initially considered by his family and primary care team due to the added risks of potential bleeding and vascular access-related complications. After discussing the matter with the patient's family, the team decided to proceed with medical treatment.

**Figure 2:**
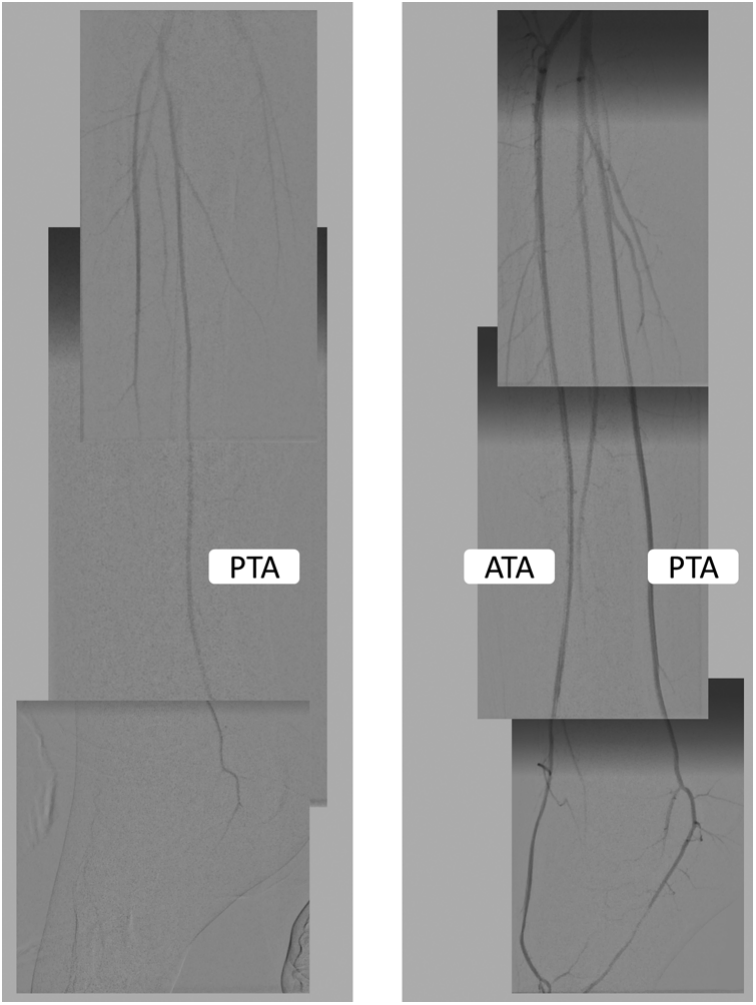
The angiography findings before and after endovascular revascularization. The left panel shows that the left anterior tibial artery flow was significantly slowed, and the flow in the posterior tibial artery did not reach the metatarsal arteries. The pedal arch was occluded. After endovascular revascularization, the flow into the anterior tibial artery and pedal arch improved.

However, on day 5 of hospitalization, there was no improvement in the left second toe cyanosis, and the resting pain in the left forefoot persisted. The treatment strategy thus was changed from medical to invasive endovascular approach. During the invasive angiography procedure, the patient's platelet count improved to 257 k/µl after receiving treatment with prednisolone and hydroxychloroquine. Vascular access was established through an ultrasound-guided puncture of the left common femoral artery, and a 6 Fr sheath was placed. The supra-popliteal and posterior tibial arteries were open, with no thrombus detected on the angiography. The flow from the origin of the anterior tibial to dorsalis pedis arteries was prolonged and sluggish (Figure 2). However, the flow stopped at the left dorsalis pedis artery, and the pedal arch to the metatarsal arteries was occluded. After placing a 6 Fr sheath, a soft-tipped 0.014-inch peripheral workhorse wire entered the left posterior tibial artery and the pedal arch (Hi-Torque Command, 300 cm, Abbott, Santa Clara, CA, USA). The smooth wire motion without resistance suggested that the obstructive nature was a soft thrombotic clot, not atherosclerosis. Angioplasty with a peripheral balloon (Amphirion 2.0 mm × 150 mm, Medtronic, Minneapolis, MN, USA) failed to re-establishing the flow. Aspiration thrombectomy then became the salvage endovascular treatment strategy. Because the clot in the distal dorsalis pedis artery and the pedal arch occluded at the plantar branch from the posterior tibial artery, we considered that a dedicated aspiration catheter used during acute ischemic stroke was most suitable in this situation.

**Figure 3:**
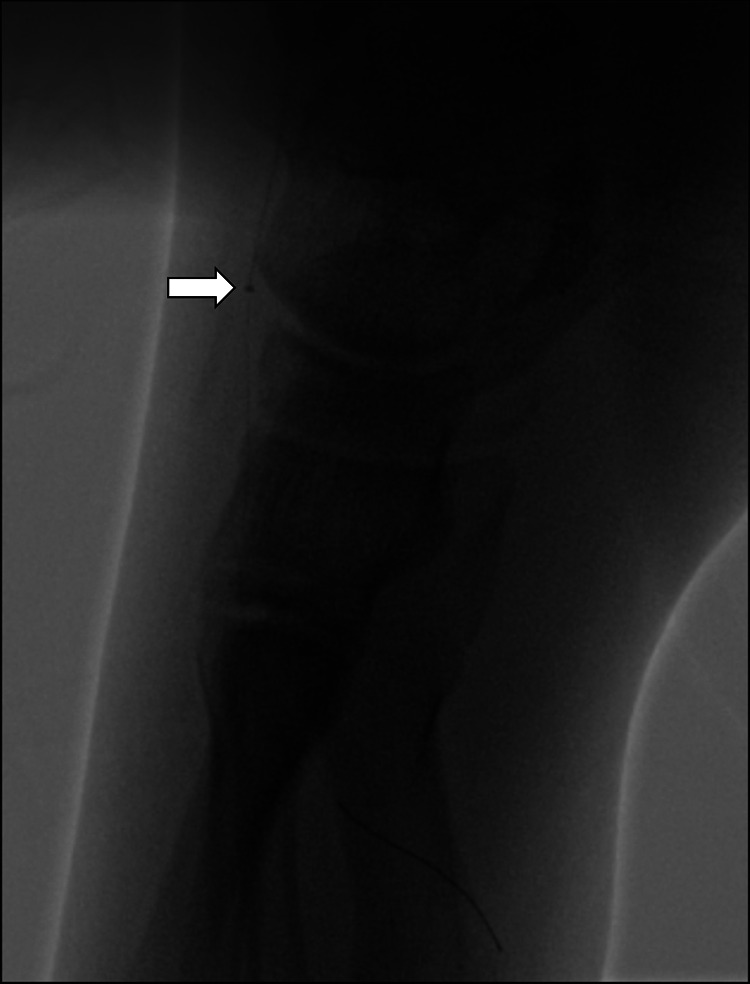
The Penumbra ACE 3Max aspiration catheter can be advanced into the left dorsalis pedis artery and pedal arch during aspiration thrombectomy (arrow). Small clots were retrieved.

We advanced Penumbra 3 Max catheter (150 cm, Penumbra, Alameda, California, USA) into the left dorsalis pedis artery while keeping continuous aspiration by Penumbra pump. After 90 s, the aspiration catheter was slowly retrieved while maintaining negative aspiration pressure (Figure 3). The flow was restored immediately after one pass of Penumbra 3 Max. Small thrombi were removed. The endovascular treatment was then completed with 180 s of balloon angioplasty to the dorsalis pedis artery-plantar arch-plantar branch of the posterior tibial artery (Amphirion 2.0 mm × 150 mm, seven Barr, for 180 s). The flow to distal metatarsal arteries improved significantly (Figure 2). There was no dissection or distal embolization. The radiation dose was 33.6 mGy, the total fluoroscopy time was 18.5 min, and a total contrast medium volume of 50 ml was used during the procedure. One hour after completion of the procedure, the sheath was removed when the activated clotting time was below 150 s. Hemostasis was achieved using manual compression for 15 min followed by sandbag compression for an additional 6 h. The patient was allowed to ambulate 8 h after the procedure. The foot pain, cyanosis, and swelling were relieved, but the gangrene change of his left second distal toe was irreversible.

**Figure 4:**
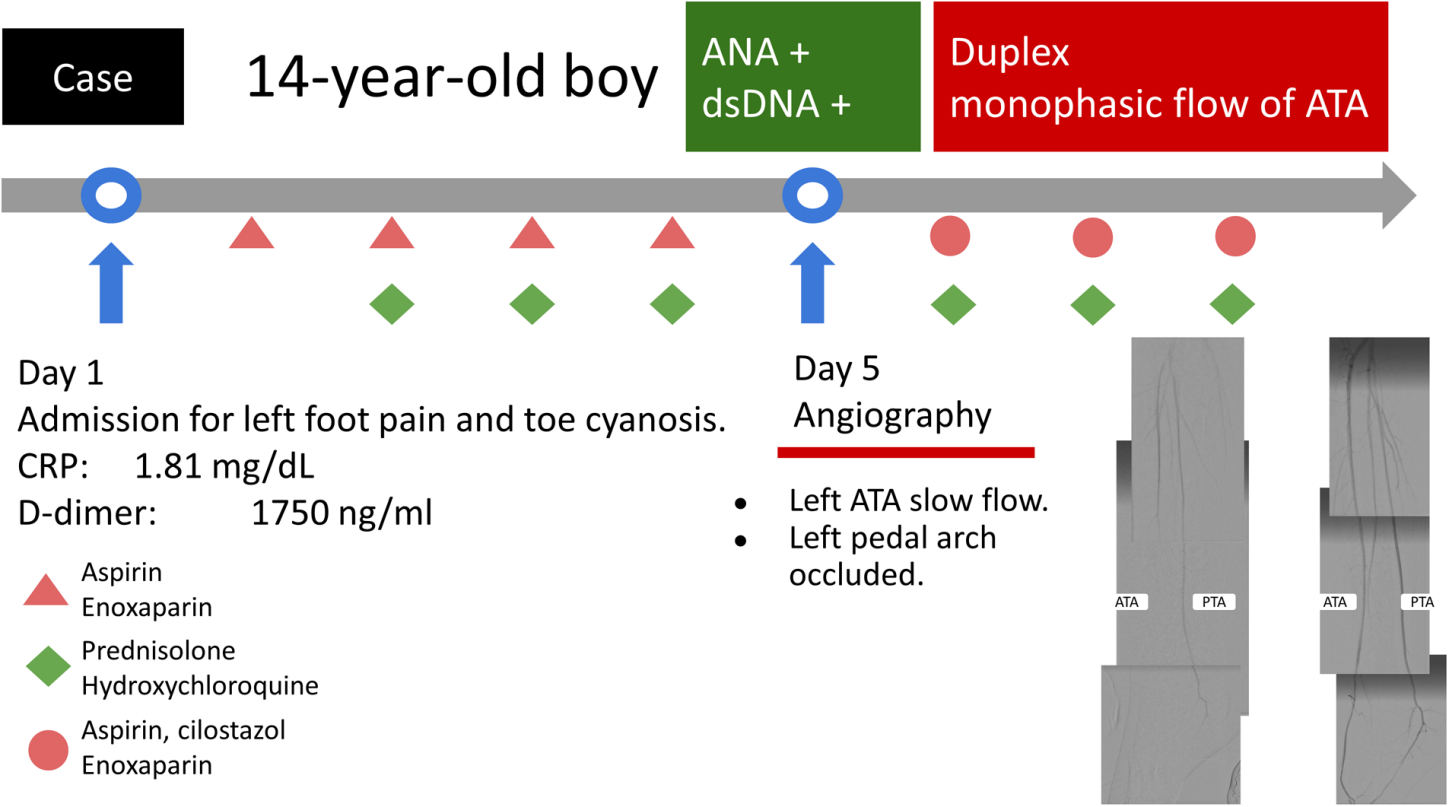
The treatment course and timeline during hospitalization.

The patient was prescribed oral dual antiplatelet agents, including aspirin 100 mg daily and cilostazol 50 mg twice daily, and underwent a 2-day course of subcutaneous enoxaparin at 1 mg/kg. The treatment course and timeline can be seen in Figure 4. However, one week after discharge, the patient developed acute respiratory distress due to pulmonary hemorrhage. As a result, urgent plasmapheresis and mini-pulse therapy with methylprednisolone were administered. The patient responded well to the treatment and was able to return to school one month after lower limb revascularization and plasmapheresis. Fortunately, toe amputation was not necessary. As for the anti-inflammatory therapy, his corticosteroid was tapered gradually, and at 9 months after the event, his medications were as follows: aspirin 100 mg/day, azathioprine 50 mg/day, hydroxycholoroquine 200 mg/day, and amlodipine 2.5 mg/day, without use of anticoagulant or steroid. At 6-month and 1-year follow-up, the autoimmune profiles including ANA, anti-dsDNA, C3, C4, and titers of anti-phospholipid antibodies were all normalized. Therefore, the pediatric rheumatologist considered anticoagulation was not necessary.

At 3 months after the index procedure, the gangrene at the patient's left 2nd toe tip spontaneously dislodged. At the one-year follow-up, the patient's five toes were preserved, and vascular Duplex showed normal flow in the left lower limb arteries. The patient was able to resume playing basketball. At the 3-year follow-up, there was no recurrence of thrombotic event.

## Discussion

In the pediatric population, acute limb ischemia is a rare occurrence. This case is unique in that, after the initial medical treatment failed, interventional devices typically used in acute stroke interventions were utilized to salvage the limb of our patient, despite the small diameter of the affected tibial artery.

Trauma with a fractured toe and soft tissue infection with cellulitis is more common in adolescents with acute foot pain with toe cyanosis. In our case, the history and physical exam all led to a correct diagnosis of peripheral artery disease. A peripheral Duplex confirmed flow abnormality in the anterior tibial artery, while the flow in the posterior tibial artery was preserved. Since initial medical treatment failed, an invasive angiography identified that the pedal arch was occluded at the forefoot level, and the patent posterior tibial artery flow could not reach the toes. In our case, non-invasive CT angiography and peripheral Duplex have limitations- they cannot comprehensively assess distal circulation. From the angiography, the stenosis in the distal plantar branch of the posterior tibial artery and occlusion in the dorsalis pedis artery contributed to poor flow into the metatarsal arteries. Ankle-brachial index and even vascular Duplex may not be able to identify the anatomical obstruction since the occlusion site is distal to ankle vessels. Only toe brachial index or subcutaneous tissue oxygen, which may not be available in a resource-limited care facility, may be able to identify lesions in the plantar or dorsalis pedis arteries. The diagnostic accuracy of contrast-enhanced computer tomography of the tibial and pedal arteries was poor because of the small vessel size. Therefore, when medical treatment fails in a patient with acute limb ischemia, it is essential to pursue a definitive invasive angiography to confirm the anatomical obstruction site and the severity of stenosis or occlusion. According to the angiographic findings, if acute limb ischemia was not identified and appropriately treated in our case, progressive gangrene change and subsequent infection involving toes and forefoot were inevitable.

In our patient, the cause of the distal anterior tibial artery and the pedal arch was related to thrombosis due to SLE and APS. In the pediatric population, the incidence of childhood-onset systemic lupus erythematosus was 0.7 per 100,000 children (2). And among antiphospholipid antibody-positive children with lupus, the annual thrombosis rates were reported between 2.2% to 6.6% (3–6). Because of its rare incidence, utilizing endovascular thrombectomy devices in pediatric population has not been reported before. Treating thrombus in the distal tibial artery extending to small plantar arteries is challenging. Balloon angioplasty alone may compromise the flow due to clot disruption with subsequent distal embolization. George et al. reported the results of using ultrasound-assisted catheter-directed thrombolysis to salvage acute limb ischemia (7). However, in our case, it would be difficult to place the infusion catheter into the dorsalis pedis artery due to the small vessel size in the pediatric population. It is especially contraindicated when the vessel lumen is smaller than the catheter diameter. In this situation, the Penumbra aspiration system (Penumbra Inc., Alameda, California), which has a soft tip to facilitate tracking of tortuous brain vessels and perform large-bore aspiration power for stroke intervention, is most suitable in this scenario. Farhat-Sabet et al. reported using Penumbra Indigo (Penumbra Inc., Alameda, California) to successfully treat iatrogenic distal embolization in four patients with acute limb ischemia (8). In the PRISM trial, the reported success rate with Penumbra Indigo aspiration catheter was 87.2% (9). In the INDIAN registry, the primary technical success rate was 88.7% in 150 patients presented with acute limb ischemia (10). But both the PRISM trial and the INDIAN registry included mainly the adult population, and the catheter used was Penumbra Indigo (a large bore catheter compared to ACE 3Max) (9). The Penumbra aspiration catheter system has a variety of choices for different catheter sizes, allowing it to advance to the M2 segment of the middle cerebral artery with its ACE 3Max catheter. In our case, the ACE 3Max aspiration catheter entered the dorsalis pedis artery, a vessel diameter of about 2 mm.

APS has a thrombotic tendency *in vivo* while having prolonged coagulation tests *in vitro*, so performing arterial puncture and endovascular intervention is challenging. We believe that adequate anti-inflammation and disease-modifying anti-rheumatic drugs are prerequisites for successful endovascular salvage when medical treatment fails to open the clotted vessel. Also, it is essential to allow early arterial sheath removal after a successful endovascular procedure.

In the pediatric population, there are limited choices in endovascular device selection, and our case report suggested a reasonable approach. However, this case report has its limitations. First, acute limb ischemia is rare in the pediatric population. A randomized controlled trial to compare the results of different treatment modalities is challenging. Second, our treatment strategy cannot be used in vessels with potential dissection and atherosclerosis.

Acute limb ischemia is a rare complication of SLE that may require urgent invasive endovascular intervention combined with medical therapy. To provide limb salvage, operators may combine peripheral and neuro-intervention devices to maximize procedure success.

## Data Availability

The original contributions presented in the study are included in the article, further inquiries can be directed to the corresponding author.

## References

[1] Lim S Javorski MJ Halandras PM Kuo PC Aulivola B Crisostomo P. Epidemiology, treatment, and outcomes of acute limb ischemia in the pediatric population. J Vasc Surg. (2018) 68:182–8. 10.1016/j.jvs.2017.11.064 29502995

[2] Valenzuela-Almada MO Hocaoglu M Dabit JY Osei-Onomah SA Basiaga ML Orandi AB Epidemiology of childhood-onset systemic lupus erythematosus: a population-based study. Arthritis Care Res (Hoboken). (2022) 74:728–32. 10.1002/acr.24827 34825516 PMC9038612

[3] Madison JA Zuo Y Knight JS. Pediatric antiphospholipid syndrome. Eur J Rheumatol. (2019) 7:1–10. 10.5152/eurjrheum.2019.19160 31804173 PMC7004270

[4] Ahluwalia J Singh S Naseem S Suri D Rawat A Gupta A Antiphospholipid antibodies in children with systemic lupus erythematosus: a long-term clinical and laboratory follow-up status study from northwest India. Rheumatol Int. (2014) 34:669–73. 10.1007/s00296-013-2736-x 23563494

[5] Descloux E Durieu I Cochat P Vital Durand D Ninet J Fabien N Paediatric systemic lupus erythematosus: prognostic impact of antiphospholipid antibodies. Rheumatology (Oxford). (2008) 47:183–7. 10.1093/rheumatology/kem335 18160418

[6] Levy DM Massicotte MP Harvey E Hebert D Silverman ED. Thromboembolism in paediatric lupus patients. Lupus. (2003) 12:741–6. 10.1191/0961203303lu458oa 14596422

[7] George EL Colvard B Ho VT Rothenberg KA Lee JT Stern JR. Real-world outcomes of EKOS ultrasound-enhanced catheter-directed thrombolysis for acute limb ischemia. Ann Vasc Surg. (2020) 66:479–85. 10.1016/j.avsg.2019.12.026 31917220

[8] Farhat-Sabet AA Tolaymat B Voit A Drucker CB Santini-Dominguez R Ucuzian AA Successful treatment of acute limb ischemia secondary to iatrogenic distal embolization using catheter directed aspiration thrombectomy. Front Surg. (2020) 7:22. 10.3389/fsurg.2020.00022 32391375 PMC7192036

[9] Saxon RR Benenati JF Teigen C Adams GL Sewall LE Trialists P. Utility of a power aspiration-based extraction technique as an initial and secondary approach in the treatment of peripheral arterial thromboembolism: results of the multicenter PRISM trial. J Vasc Interv Radiol. (2018) 29:92–100. 10.1016/j.jvir.2017.08.019 29128156

[10] de Donato G Pasqui E Sponza M Intrieri F Spinazzola A Silingardi R Safety and efficacy of vacuum assisted thrombo-aspiration in patients with acute lower limb ischaemia: the INDIAN trial. Eur J Vasc Endovasc Surg. (2021) 61:820–8. 10.1016/j.ejvs.2021.01.004 33648846

